# Effects of Dynamic Neuromuscular Stabilization on Lower Limb Muscle Activity, Pain, and Disability in Individuals with Chronic Low Back Pain: A Randomized Controlled Trial

**DOI:** 10.3390/medicina61111961

**Published:** 2025-10-31

**Authors:** Farhad Rezazadeh, Shirin Aali, Fariborz Imani, Hamed Sheikhalizadeh, Ibrahim Ouergui, Razvan-Sandu Enoiu, Luca Paolo Ardigò, Georgian Badicu

**Affiliations:** 1 Department of Sports Biomechanics, Faculty of Educational Sciences and Psychology, University of Mohaghegh Ardabili, Ardabil P.O. Box 56199-11367, Iran; rezazadeh.farhad@uma.ac.ir (F.R.); fariborzimani@uma.ac.ir (F.I.); hamed.sh@uma.ac.ir (H.S.); 2 Department of Sport Science Education, Farhangian University, Tehran P.O. Box 889-14665, Iran; 3 High Institute of Sport and Physical Education of Kef, University of Jendouba, Kef 7100, Tunisia; brahim.ouerghi@issepkef.u-jendouba.tn; 4 Research Unit, Sport Sciences, Health and Movement, UR22JS01, University of Jendouba, Kef 7100, Tunisia; 5 Department of Motor Performance, Transilvania University of Brasov, 500068 Brasov, Romania; razvan.enoiu@unitbv.ro; 6 Department of Teacher Education, NLA University College, 0166 Oslo, Norway; 7 Department of Physical Education and Special Motricity, Faculty of Physical Education and Mountain Sports, Transilvania University of Braşov, 500068 Braşov, Romania; georgian.badicu@unitbv.ro

**Keywords:** chronic low back pain, Dynamic Neuromuscular Stabilization, electromyography, gait, motor control, rehabilitation

## Abstract

*Background and Objectives:* Chronic low back pain (CLBP) is associated with altered neuromuscular control. Dynamic Neuromuscular Stabilization (DNS) targets core–limb coordination; however, its specific impact on lower-limb electromyographic (EMG) activity during gait remains unclear. *Materials and Methods:* Fifty-five young adults with non-specific CLBP (pain ≥ 3 months with no identifiable specific pathology) completed the trial (overall mean age 23.7 ± 1.3 years). Participants were randomized to an 8-week DNS program or a control. Pre-/Post-intervention surface EMG during gait and clinical outcomes (VAS, ODI) were assessed. *Results:* Compared with control, DNS showed lower adjusted Post-test VAS (3.08 ± 0.25 vs. 6.13 ± 0.24; *ηp*^2^ = 0.596) and ODI (15.73 ± 1.55% vs. 34.36 ± 1.52%; *ηp*^2^ = 0.579). Directionally, DNS was associated with phase-specific EMG modulation: tibialis anterior during mid-stance was lower (*ηp*^2^ = 0.137), rectus femoris during push-off was lower (*ηp*^2^ = 0.119), biceps femoris during push-off was lower (*ηp*^2^ = 0.168), and vastus medialis at heel-strike was higher (*ηp*^2^ = 0.077) relative to control. Other muscle–phase pairs showed no adjusted between-group differences. *Conclusions:* An 8-week DNS program was associated with clinically meaningful reductions in pain and disability and with phase-specific changes in lower-limb EMG during gait. These findings support DNS as a promising rehabilitation option for young adults with CLBP; confirmation in larger trials with active comparators is warranted.

## 1. Introduction

CLBP is among the most prevalent musculoskeletal problems, with approximately 80% of individuals experiencing at least one episode during their lifetime [1]. Although low back pain (LBP) is often considered a self-limiting condition, long-term follow-up indicates that about 33% of affected individuals report moderate pain and 15% severe pain one year after the initial episode [2]. In addition to persistent discomfort, patients with CLBP commonly present with motor control impairments, particularly involving the lower limbs, which may contribute to the chronicity and recurrence of symptoms [3]. These impairments are often accompanied by postural instability and aberrant trunk kinematics during functional activities such as standing, sitting, and ambulation [4,5]. Such findings highlight the critical role of neuromuscular function in maintaining postural control and efficient movement, underscoring the importance of targeted rehabilitation strategies.

Pain-induced alterations in neuromuscular coordination can lead to maladaptive movement patterns and dysfunctional recruitment of musculature [6]. For example, surface EMG studies have demonstrated abnormal activation patterns in key trunk muscles, including the erector spinae and external oblique, in individuals with CLBP compared to healthy controls [6,7,8]. Such findings highlight the critical role of neuromuscular function in maintaining postural control and efficient movement, underscoring the importance of targeted rehabilitation strategies. Given these motor-control impairments, interventions that restore trunk–limb coordination may translate into more efficient gait behavior in individuals with CLBP.

Efficient human movement depends on the coordinated activation of multiple muscle groups to maintain postural stability and execute dynamic tasks [9]. Individuals with CLBP often exhibit impaired movement control characterized by delayed muscle activation, reduced precision in trunk motions, and altered mechanical stiffness of the lumbo-pelvic region [10,11,12]. While these neuromuscular adaptations may serve as short-term compensatory strategies to minimize pain during activity, they frequently result in inefficient movement patterns and may reinforce the chronic pain cycle [13].

Building on this rationale, a growing body of evidence supports the existence of a functional kinetic chain between the trunk and lower extremities, wherein the trunk serves as a central conduit for the generation and distribution of forces throughout the body during locomotion [14]. Disruptions in this kinetic relationship can negatively impact motor control and gait efficiency. Empirical work has linked altered trunk mechanics with downstream changes in lower-extremity control during locomotion and balance tasks [10,14]. In individuals with CLBP, such linkages may amplify gait inefficiencies through suboptimal coordination strategies, suggesting that interventions targeting integrated trunk stabilization could plausibly influence gait-phase–specific activation patterns in the lower limb during walking [15,16]. Against this biomechanical backdrop, a motor control–oriented approach that prioritizes coordinated trunk–limb stabilization is theoretically well positioned to address these deficits.

Against this biomechanical backdrop, a motor control–oriented approach that prioritizes coordinated trunk–limb stabilization is theoretically well positioned to address these deficits. DNS aims to reintegrate optimal core-limb coordination by enhancing motor control and restoring developmental movement patterns [15,17]. Through targeted activation of deep stabilizing muscles, DNS is thought to facilitate more efficient force transmission and reduce mechanical stress on the musculoskeletal system during gait. Core strength is therefore not only a stabilizing factor but also a key contributor to dynamic force regulation across the kinetic chain [18]. Consequently, identifying and implementing interventions that effectively restore neuromuscular control remains a crucial objective in the management of CLBP.

In recent years, therapeutic strategies for managing LBP have increasingly emphasized exercises that enhance local spinal stability, particularly through proprioceptive retraining of the lumbo-pelvic region [19]. Central to this approach is the targeted activation of deep abdominal muscles, which are integral to segmental stabilization of the lumbar spine and play a key role in maintaining neuromuscular control during functional movements. One such intervention is DNS, a motor control-based method that draws upon the principles of developmental kinesiology. DNS utilizes postural and movement patterns observed in early infancy to assess and rehabilitate dysfunctions in motor coordination and joint stabilization [16].

Emerging evidence suggests that neuromuscular impairments, such as delayed muscle recruitment or faulty motor sequencing, may underlie biomechanical dysfunctions commonly seen in individuals with chronic musculoskeletal pain, including LBP [6,10,13]. These deficits are associated with reduced postural stability and suboptimal coordination strategies, which can compromise joint control and contribute to symptom persistence or recurrence [6,11,12,13]. Such neuromuscular impairments may be mitigated through targeted rehabilitation strategies aimed at restoring motor control and stability. The clinical evidence supporting DNS efficacy specifically for low back pain is still developing. These observations align with prior clinical reports indicating DNS-related improvements in neuromuscular control among individuals with LBP [20].

A recent 2025 randomized controlled trial combining DNS with Kinesio Taping reported improvements in core-muscle activation and pain self-efficacy in adults with chronic non-specific low back pain [21]. This six-week intervention assessed multifidus and transversus abdominis using surface EMG. Although gait parameters were not evaluated, improved core activation offers a plausible pathway through which DNS might influence walking. A recent scoping review identified only four studies examining DNS in chronic low back pain, indicating a limited but growing evidence base with promising neuromuscular findings [22]. To date, no studies have directly examined DNS-related changes in lower-limb EMG during gait, representing a critical gap; likewise, the effectiveness of DNS on fundamental locomotor tasks has not been thoroughly investigated [16]. Complementary randomized and controlled clinical trials have reported reductions in pain and disability following DNS-based programs [23,24,25,26]. Collectively, these observations motivated the current trial to test whether an 8-week DNS program is associated with gait-phase–specific changes in lower-limb EMG and improvements in pain and disability.

Although DNS has gained increasing attention as an intervention to enhance core stability and neuromuscular coordination, evidence regarding its effects in individuals with CLBP remains limited. Notably, no prior studies have directly examined the influence of DNS on lower-limb muscle activity during gait—a fundamental functional task frequently impaired in this population. The primary objective of this trial was to determine whether an eight-week DNS program would be associated with phase-specific modulation of lower-limb surface EMG during over ground gait, along with reductions in pain and disability in young adults with chronic non-specific. We hypothesized that DNS would improve lower-limb muscle activation patterns and reduce pain and disability compared with controls, with analyses adjusted for baseline values.

## 2. Materials and Methods

### 2.1. Participants and Design

This randomized controlled trial recruited 60 young adults (28 men, 27 women) with non-specific CLBP aged 21–25 years in Ardabil, Iran. The overall mean age of the sample was 23.7 ± 1.3 years. Table 1 summarizes baseline demographic and anthropometric characteristics. At baseline, the DNS group (*n* = 27) had an age of 23.9 ± 1.3 years, height 175.2 ± 5.4 cm, weight 74.2 ± 7.2 kg, and BMI 24.1 ± 2.0 kg/m^2^. The Control group (*n* = 28) had an age of 23.6 ± 1.4 years, height 174.4 ± 6.4 cm, weight 74.2 ± 7.6 kg, and BMI 24.3 ± 2.1 kg/m^2^. Sex distribution was balanced in both groups (DNS 14/13; Control 14/14 see Table 1), and no statistically significant between-group differences were observed at baseline (all *p* > 0.05; see Table 2). The sample size was calculated based on a previous study investigating EMG changes in LBP populations [23], using an effect size of 0.30 (Cohen’s f; moderate effect), a significance level of α = 0.05, and a statistical power of 0.85 via G*Power software (version 3.1) [27]. A minimum total sample of 28 participants (14 per group) was determined; to enhance precision, allow for sex-balanced enrollment, and accommodate anticipated attrition, the sample was increased to 60 (30 per group). Participants were recruited from a specialized medical clinic and diagnosed with non-specific CLBP by a neurologist.

**Figure 1:**
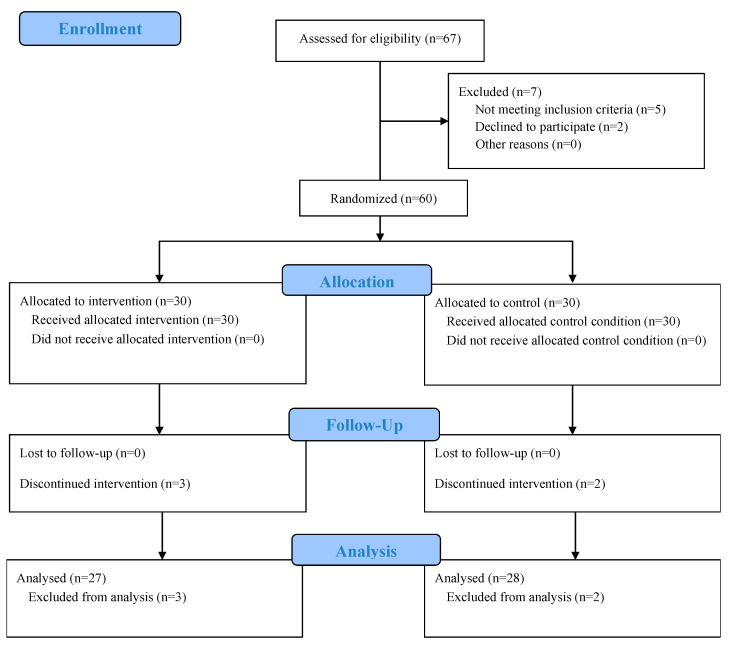
CONSORT flow diagram showing numbers screened, excluded with reasons (not meeting inclusion, declined to participate, other), randomized, allocated to DNS/control, lost to follow-up with reasons, and included in analysis.

At baseline, participants reported an average pain duration of 11.4 ± 3.2 months, consistent with the inclusion criterion of ≥3 months for chronicity. All individuals had no prior structured rehabilitation program for LBP and were not taking analgesic or anti-inflammatory medication during the study period. Pain severity at enrollment corresponded to moderate intensity (VAS range 4–8), and ODI scores (30–40%) indicated a moderate level of functional limitation. Based on screening interviews, participants were untrained university students with no regular exercise routine during the past six months, and their occupations were sedentary or academic in nature. None reported participation in competitive or professional sports. Figure 1 presents the CONSORT flow diagram of screening, randomization, and analysis. 

### 2.2. Randomization and Blinding

Participants were randomized in a 1:1 ratio using a computer-generated sequence (Excel RAND) prepared by an independent coordinator with no role in enrollment or outcome assessment. Allocation was centrally administered: after eligibility confirmation and completion of all baseline measurements, the coordinator released the next sequential assignment to the treating therapist. Investigators responsible for enrollment and the outcome assessor had no access to the randomization list. The assessor who performed sEMG acquisition and administered VAS/ODI remained blinded to group allocation throughout all study visits, and participants were instructed not to disclose their group assignment during assessments. Due to the nature of the intervention, therapists and participants could not be blinded; however, standardized measurement protocols and scripted instructions were implemented to minimize assessment bias. No stratification was performed, which may introduce a small risk of baseline imbalance. During the intervention, five participants discontinued (DNS: *n* = 3; Control: *n* = 2), leaving 55 participants for analysis (DNS: *n* = 27; Control: *n* = 28; DNS men/women 14/13; Control men/women 14/14; see Figure 1).

### 2.3. Inclusion and Exclusion Criteria

The inclusion criteria for participation in this study were: having a minimum of three months of non-specific CLBP, the ability to sit, stand, and walk without using assistive devices, not engaging in regular exercise, no history of musculoskeletal surgery involving the trunk or lower limbs, no presence of neuromuscular or orthopedic conditions, no limb length discrepancies exceeding 5 mm, and no activity limitations as advised by a healthcare professional [28]. The exclusion criteria included having any specific pathology, such as disc protrusion, spinal injury, or surgery history; neurological disorders; tumors; osteoarthritis; and rheumatoid arthritis diagnosed by a specialist physician; being diagnosed with any medical condition that restricts physical activity by a physician; experiencing unbearable pain and an inability to perform exercises; absenteeism of more than one-third of exercise sessions irregularly or absenteeism of three consecutive sessions; and a lack of willingness to continue participation in the study [28]. Both men and women were eligible to enhance generalizability and capture potential sex-related variability. The study procedures were fully explained to participants, and informed written consent was obtained per the Declaration of Helsinki [29]. Demographic data such as age, limb dominance, and injury history were collected, with limb dominance determined by standardized ball kicking and throwing tests. The research approach was registered in the Iranian Registry of Clinical Trials (IRCT20240917063072N2).

### 2.4. Intervention (DNS Protocol)

The subjects were randomly assigned to two groups: a treatment group (Group 1, *n* = 30; analyzed *n* = 27) and a control group (Group 2, *n* = 30; analyzed *n* = 28). The subjects in the treatment group participated in the DNS exercise program for 8 weeks (50 min, 3 sessions per week), while the control group did not perform any structured exercise and was re-evaluated after 8 weeks. All participants were asked not to participate in other sports and activities during the intervention period. Participants in the control group were instructed to maintain their usual daily activities and were explicitly asked not to begin any new exercise programs during the study period. The intervention included two core elements: theoretical instruction and practical training. The theoretical session consisted of a two-hour class focused on teaching participants proper techniques for performing functional daily movements. The DNS exercise protocol in this study was adapted from the work of Zolaktaf et al. (2020) [16]. Accordingly, the control group did not perform the exercise program, whereas the treatment group followed a DNS exercise protocol for a whole period of 8 weeks (three 50 min sessions per week). The DNS group’s protocol involved a 5 min warm-up, 40 min of DNS movements accompanied by breathing exercises, and a 5 min cool-down. Exercise intensity was standardized using the Borg CR10 scale. Participants maintained a perceived exertion of 3–5 (moderate) during the ~40 min DNS block. RPE was recorded in the final minute of each position/set and used to guide weekly progression (e.g., adding a task or extending hold duration while remaining within the target RPE). All sessions were supervised by licensed physiotherapists at an approximate therapist-to-participant ratio of 1:6 during group sessions. Therapists provided continuous verbal/tactile cueing, verified diaphragmatic breathing and alignment before progression, and documented RPE and exercises completed in session logs. Supervision was sex-matched: female participants were supervised by female physiotherapists, and male participants were supervised by male physiotherapists. Group sessions were organized accordingly to preserve the ~1:6 therapist-to-participant ratio within same-sex groups. According to the DNS approach [15,16], the exercises included diaphragmatic breathing, Baby Rock (supine 90–90), prone, rolling, side lying, oblique sit, tripod, kneeling, squat, and Czech Get Up (CGU) (Appendix A.1). Visual exemplars and therapist cueing points for these positions are shown in Appendix A.2. Week one specifically involved training and practicing basic DNS exercises. The complexity of the exercises increased gradually by adding a new task to an already practiced task every week (in comparison with the preceding week). In the first session, the trainer demonstrated the correct execution of each position and provided relevant explanations. Afterward, all participants performed the exercise protocol, and the trainer supervised their proper execution. The outcome assessor was blinded to group allocation. A photo-plate illustrating session flow and key DNS positions is provided in Appendix A.2 (panels A–H), including alignment and breathing checkpoints for each position. Over 8 weeks (24 planned sessions), the DNS group (*n* = 27) completed 638/648 sessions (98.5% attendance; mean 23.6 sessions per participant) with small-group delivery at an approximate therapist-to-participant ratio of 1:6, and no adverse events leading to withdrawal were recorded.

### 2.5. EMG Acquisition and Processing

The EMG data were obtained from the tibialis anterior (TA), gastrocnemius (GC), Vastus Lateralis (VL), Vastus medialis (VM), rectus femoris (RF), biceps femoris (BF), semitendinosus (ST) and gluteus medius (GM) muscles of the dominant lower limb using the 8-channel electromyographic device (DataLITE EMG, Biometrics Ltd., Bandwidth: 10–490 HZ, Newport, UK) during three gait trials [30,31]. Gait speed was measured over the central 10 m of a 15 m walkway using a handheld stopwatch, following the standard 10-Meter Walk Test protocol to ensure consistent self-selected walking velocity [32]. Surface EMG data were recorded from the tibialis anterior (TA), gastrocnemius (GC), vastus lateralis (VL), vastus medialis (VM), rectus femoris (RF), biceps femoris (BF), semitendinosus (ST), and gluteus medius (GM) of the dominant lower limb using an 8-channel system (DataLITE EMG, Biometrics Ltd., 10–490 Hz, UK) during overground walking [30,31]. Participants completed three gait trials over a 15 m walkway [32]. After establishing a comfortable self-selected pace, cadence and speed were stabilized using an auditory metronome based on each participant’s baseline cadence. Walking speed was measured over the central 10 m, and trials were repeated if cadence or speed deviated by more than ±5%. EMG amplitudes were averaged across the three accepted trials for each gait phase (loading response, mid-stance, push-off, swing) and normalized to maximum voluntary contraction (MVC). The number of trials (*n* = 3) balanced measurement precision and participant burden [32]. Intra-trial reliability was high (ICC = 0.82–0.94), confirming consistency across trials.

Before sensor application, each muscle was palpated during isometric contraction, the skin surface overlying the center of the muscle belly was cleaned with alcohol, and the hair was removed. Sensors were attached to the skin using hypoallergenic double-sided tape with an inter-electrode distance of 20 mm. The specific locations for sensor placement were determined according to the Surface Electromyography for Non-Invasive Assessment of Muscles (SENIAM) guidelines, as detailed in Table 2 [33,34]. The recorded raw EMG data underwent a comprehensive processing and amplitude analysis protocol to quantify muscle activation during each gait phase [34,35]. First, the raw EMG signals were filtered using a 6th-order Butterworth bandpass filter with a cutoff frequency range of 10–450 Hz to remove noise and movement artifacts. Following this, the filtered signal was full-wave rectified. To create a smooth representation of the muscle activation profile, a linear envelope was generated by applying a low-pass 6 Hz Butterworth filter to the rectified signal [35]. The resulting linear envelope represents the amplitude of the EMG signal over time. The mean amplitude of this signal was then calculated for each distinct phase of the gait cycle (Loading Response (0–20%), Mid-Stance (20–40%), Push-Off (40–60%), and Swing (60–100%)). Gait sub-phases were determined using footswitch sensors (DataLITE EMG, Biometrics Ltd., UK) placed under the heel and forefoot. Foot contact and toe-off events were identified through signal changes in the footswitch, and all EMG and footswitch data were synchronized and analyzed using DataLITE software (version 9.0) and MATLAB R2021b [36].

### 2.6. Outcome Measures

To normalize the EMG signals, maximal voluntary isometric contractions (MVICs) were recorded for each muscle before the gait analysis, following standardized procedures against manual resistance. For each test, participants were instructed to gradually build to a maximal contraction over 3 s and hold it for 5 s. Three trials were performed for each muscle, with a 2 min rest period between trials to prevent fatigue [37]. The specific testing positions were standardized to elicit maximal activation for each muscle. For the Gluteus Medius (GM), the participant was tested in a side-lying position performing hip abduction. The hamstrings (Biceps Femoris and Semitendinosus) were tested in a prone position with the knee flexed to approximately 60 degrees, resisting knee flexion. The quadriceps group (Rectus Femoris, Vastus Lateralis, and Vastus Medialis) was assessed in a seated position with the knee flexed to 90 degrees, resisting knee extension. The Tibialis Anterior (TA) was tested in a seated position with the ankle in a neutral position, resisting dorsiflexion, while the Gastrocnemius (GC) was tested in a standing, single-leg heel raise position to achieve maximal plantarflexion [33]. The highest recorded EMG amplitude from a 1 s window during the MVIC trials was used as the reference value for normalization. During data processing, the EMG amplitude recorded during each gait phase was expressed as a percentage of this MVIC value (%MVIC).

In addition to electromyographic data, clinical outcomes were assessed both before and after the intervention. Pain intensity was evaluated using a 10 cm Visual Analogue Scale (VAS), where 0 indicated “no pain” and 10 represented “the worst imaginable pain.” Functional disability related to low back pain was measured using the Oswestry Disability Index (ODI). The ODI questionnaire consists of 10 sections, and the final score is expressed as a percentage, with higher scores indicating a greater level of disability.

### 2.7. Statistical Analysis

Normality was checked with the Shapiro–Wilk test and homogeneity with Levene’s test. Primary between-group inferences at Post-test were performed using analysis of covariance (ANCOVA) with Group (DNS vs. Control) as the fixed factor and the corresponding Pre-test value entered as the covariate (Type III SS). For clinical outcomes (VAS, ODI), adjusted means (EMMs ± SE), F, *p*, and partial eta-squared (*ηp*^2^) are reported; covariate terms are shown for completeness. For EMG, separate ANCOVAs were run for pre-specified muscle–phase pairs; false discovery rate (FDR) control (Benjamini–Hochberg) was applied across these comparisons. Sensitivity analyses additionally adjusted for sex and BMI as covariates; inferences were unchanged and are not tabulated. Two-sided α = 0.05. Analyses were conducted in SPSS v26 (SPSS Inc., Chicago, IL, USA) [38,39].

## 3. Results

### 3.1. Participants

A total of 60 participants with non-specific chronic low back pain (28 men, 27 women) were randomized to the DNS group (*n* = 30) or the Control group (*n* = 30). During the intervention, five participants discontinued (DNS: *n* = 3; Control: *n* = 2), leaving 55 participants for analysis (DNS: *n* = 27; Control: *n* = 28; DNS men/women 14/13; Control men/women 14/14). No significant between-group differences were observed at baseline for age, height, weight, BMI, or sex distribution (all *p* > 0.05), indicating successful randomization and comparable groups (Section 2.1; Figure 1 and Table 2).

### 3.2. Clinical Outcomes

Using ANCOVA with the Post-test as the dependent variable and the corresponding pre-test as the covariate, the DNS group showed significantly lower pain and disability than the Control group after adjustment for baseline. For VAS, adjusted means (EMMs) were DNS = 3.08 ± 0.25 and Control = 6.13 ± 0.24; the Group effect was significant (F(1, 52) = 76.66, *p* < 0.001, *ηp*^2^ = 0.596), and Levene’s test indicated equal error variances (*p* = 0.976). For ODI, adjusted means were DNS = 15.73 ± 1.55 and Control = 34.36 ± 1.52; the Group effect was significant (F(1, 52) = 71.47, *p* < 0.001, *ηp*^2^ = 0.579), while Levene’s test suggested unequal variances (*p* < 0.001). Covariates were not significant in either model (VAS *p* = 0.560; ODI *p* = 0.359). Overall, after adjusting for baseline, DNS produced markedly greater clinical improvements than Control. Clinical outcomes (VAS, ODI) are summarized separately in Table 3.

### 3.3. EMG Outcomes

At baseline, no statistically significant between-group differences were observed across tibialis anterior (TA), gastrocnemius (GC), vastus lateralis and medialis (VL/VM), rectus femoris (RF), biceps femoris (BF), semitendinosus (ST), or gluteus medius (GM) in any gait phase (all *p* ≥ 0.05). The smallest *p*-values were observed for RF during swing (*p* = 0.090) and VM during swing (*p* = 0.091), but these did not reach significance. Therefore, subsequent group-by-time effects cannot be attributed to baseline imbalances. Accordingly, primary analyses used ANCOVA with the corresponding baseline value entered as a covariate (Table 4).

Following baseline checks, post-intervention between-group differences were evaluated using ANCOVA with the corresponding baseline value entered as a covariate. Adjusted Post-test means indicated markedly greater improvements in clinical outcomes for DNS versus control: pain intensity (VAS) (F(1, 52) = 76.66, *p* < 0.001, *ηp*^2^ = 0.596; adjusted means: DNS 3.08 ± 0.25, Control 6.13 ± 0.24) and disability (ODI) (F(1, 52) = 71.47, *p* < 0.001, *ηp*^2^ = 0.579; DNS 15.73 ± 1.55, Control 34.36 ± 1.52). For sEMG amplitudes, significant adjusted group effects were observed for VM during loading response (F(1, 52) = 4.35, *p* = 0.042, *ηp*^2^ = 0.077; DNS > Control), TA during mid-stance (F(1, 52) = 8.26, *p* = 0.006, *ηp*^2^ = 0.137; DNS < Control), RF during push-off (F(1, 52) = 7.02, *p* = 0.011, *ηp*^2^ = 0.119; DNS < Control), and BF during push-off (F(1, 52) = 10.49, *p* = 0.002, *ηp*^2^ = 0.168; DNS < Control). Other muscles/phases did not show significant adjusted between-group differences (*p* > 0.05). Collectively, these ANCOVA findings confirm superior clinical improvement in the DNS group, alongside phase-specific modulation of quadriceps/hamstring activity at Post-test.

Because Table 5 presents between-group comparisons at Post-test while adjusting for baseline (ANCOVA), no group × time interaction or within-group Pre-Post tests are modeled in this table. Instead, inference focuses on adjusted group effects. Significant group differences favored DNS for tibialis anterior during mid-stance (lower activation in DNS), rectus femoris during push-off (lower in DNS), and biceps femoris during push-off (lower in DNS), whereas vastus medialis during heel strike was higher in DNS than control. Other muscles/phases were not significant (*p* > 0.05). 

## 4. Discussion

This study aimed to investigate the effects of an 8-week DNS exercise program on the EMG activity of lower-limb muscles during gait in young adults with chronic non-specific CLBP. The key finding of this research is that the DNS intervention was associated with statistically significant and functionally relevant between-group differences at Post-test (ANCOVA, adjusted for baseline) at specific muscle–phase combinations. In particular, adjusted group effects favored DNS for tibialis anterior (TA) during mid-stance (MC) (F(1, 52) = 8.26, *p* = 0.006, *ηp*^2^ = 0.137), rectus femoris (RF) during push-off (PO) (F = 7.02, *p* = 0.011, *ηp*^2^ = 0.119), and biceps femoris (BF) during push-off (PO) (F = 10.49, *p* = 0.002, *ηp*^2^ = 0.168), while vastus medialis (VM) at heel strike (LR) was higher in DNS (F = 4.35, *p* = 0.042, *ηp*^2^ = 0.077) relative to control. Clinically, these patterns coincided with markedly lower pain and disability in DNS vs. control after adjustment (VAS EMMs 3.08 ± 0.25 vs. 6.13 ± 0.24; F = 76.66, *p* < 0.001, *ηp*^2^ = 0.596; ODI 15.73 ± 1.55% vs. 34.36 ± 1.52%; F = 71.47, *p* < 0.001, *ηp*^2^ = 0.579). Taken together, the findings may suggest that a centrally focused intervention like DNS is associated with phase-specific differences in distal activation during walking; however, because no core muscle or biomechanical parameters were recorded, such interpretations are correlational and hypothesis-generating rather than mechanistic.

To contextualize the statistical findings, we quantified the magnitude of the significant muscle–phase effects using partial eta-squared, and we summarized adjusted Post-test differences in %MVIC. By conventional benchmarks (partial eta-squared of approximately 0.06, indicating a medium effect, and approximately 0.14, indicating a large effect) [39], vastus medialis at heel strike showed a small-to-medium effect (*ηp*^2^ = 0.077), tibialis anterior at mid-stance a medium-to-large effect (*ηp*^2^ = 0.137), rectus femoris at push-off a medium effect (*ηp*^2^ = 0.119), and biceps femoris at push-off a large effect (*ηp*^2^ = 0.168). Based on adjusted means, tibialis anterior at mid-stance was 78.5%MVIC in the DNS group versus 95.2%MVIC in the control group (difference 16.7%MVIC, approximately 17.5 percent lower relative to control); rectus femoris at push-off was 65.6 versus 85.2%MVIC (difference 19.6%MVIC, approximately 23.0 percent lower); biceps femoris at push-off was 68.0 versus 84.9%MVIC (difference 16.9%MVIC, approximately 19.9 percent lower); and vastus medialis at heel strike was 80.7 versus 68.1%MVIC (difference 12.6%MVIC, approximately 18.5 percent higher). For clinical outcomes, between-group contrasts at Post-test were large: VAS adjusted means were 3.08 versus 6.13 (partial *ηp*^2^ = 0.596) and ODI adjusted means were 15.73 percent versus 34.36 percent (partial *ηp*^2^ = 0.579). These values are consistent with improvements that exceed commonly reported minimal clinically important differences for low back pain outcomes, indicating that the observed changes are likely to be clinically meaningful in this cohort. These magnitudes frame the associations reported above and motivate the subsequent hypothesis-generating discussion of potential mechanisms and limitations.

The observed alterations in EMG activity in the DNS group are unlikely to be incidental and may reflect a phase-specific reorganization toward more efficient motor strategies, in line with the therapeutic objectives of the DNS approach [40]. Notably, TA at mid-stance showed lower adjusted activation in DNS (*ηp*^2^ = 0.137, *p* = 0.006), RF at push-off (*ηp*^2^ = 0.119, *p* = 0.011), and BF at push-off (*ηp*^2^ = 0.168, *p* = 0.002) were also lower, whereas VM at heel strike was higher (*ηp*^2^ = 0.077, *p* = 0.042) compared with the control. This pattern is consistent with more task-appropriate coordination during load acceptance (VM at LR), mid-stance progression (TA), and propulsion (RF/BF at PO); causality cannot be inferred from the present data.

The adjusted Post-test EMG patterns are generally consistent with task-phase–specific muscle roles and efficient recruitment. Increased vastus medialis activation at heel strike aligns with the knee stabilization demands during early stance, whereas reduced tibialis anterior activity at mid-stance may reflect lower dorsiflexor requirements as the shank advances over the foot. Similarly, decreased rectus femoris and biceps femoris activation at push-off is compatible with more selective propulsion and reduced co-contraction. Conversely, the lack of significant changes in several muscle–phase pairs may reflect small or phase-unspecific effects, task constraints inherent to steady-speed overground walking, redundancy in joint moment generation across muscles, or methodological considerations such as %MVIC normalization and FDR correction. These interpretations are correlational; core muscle activity and three-dimensional biomechanical parameters were not assessed. Therefore, the proposed mechanisms remain hypothesis-generating and require confirmation in future studies.

Overall, the findings suggest that an eight-week DNS program may be associated with phase-specific alterations in distal lower-limb muscle activation during walking. Within the DNS framework, such adaptations are commonly hypothesized to reflect improvements in central stabilization, with DNS potentially influencing the integrated spinal stabilization system [15]. This system involves deep core muscles, including the diaphragm, transversus abdominis (TrA), and lumbar multifidus, which contribute to intra-abdominal pressure regulation [40]. Although previous studies indicate that DNS may affect diaphragm contractility and IAP [21,28], we did not directly measure core muscle activity or IAP. Therefore, any interpretation of distal EMG changes as mediated by core mechanisms remains speculative and correlational and should be considered a hypothesis for future research.

In aggregate, the phase-specific sEMG differences observed here are compatible with a shift toward more economical, task-appropriate recruitment rather than non-specific co-contraction patterns sometimes reported in CLBP. Notably, several muscles showed significant adjusted group effects at Post-test—tibialis anterior at mid-stance (F(1, 52) = 8.26, *p* = 0.006, *ηp*^2^ = 0.137), rectus femoris at push-off (F = 7.02, *p* = 0.011, *ηp*^2^ = 0.119), biceps femoris at push-off (F = 10.49, *p* = 0.002, *ηp*^2^ = 0.168), and vastus medialis at heel strike (F = 4.35, *p* = 0.042, *ηp*^2^ = 0.077), whereas other muscle–phase pairs did not differ after baseline adjustment. The heterogeneity of these effects—ranging from small-to-medium (VM at LR, *ηp*^2^ = 0.077) to medium-to-large (TA at MC; RF/BF at PO, *ηp*^2^ = 0.12–0.17), may reflect both phase-specific functional roles and sample-size constraints that limit power for smaller effects [38]. This pattern underscores the complexity of neuromuscular adaptation. While DNS training is intended to restore fundamental motor patterns and optimize the integrated spinal stabilization system [15,40]. Any influence on distal activation observed here should be interpreted as associative; larger studies with direct trunk measures are required to clarify mechanisms [16].

The significant adjusted difference for VM at heel strike (F = 4.35, *p* = 0.042, *ηp*^2^ = 0.077) may indicate subtle but potentially meaningful modulation of early stance knee extensor contribution. Although within-group post hoc changes were not modeled (given our ANCOVA focus on adjusted Post-test between-group effects), the small-to-medium effect size suggests a trend worthy of confirmation in larger cohorts or with longer interventions. Clinically, these findings highlight that DNS may initiate early adjustments in quadriceps-related recruitment during gait, meriting further evaluation with extended follow-up [20]. Prior work shows that altered activation timing and distribution can relate to performance and pain reductions [41,42,43]. Here, the adjusted between-group differences provide hypothesis-generating evidence for such links.

Our findings build on previous studies demonstrating that DNS can reduce pain and disability and improve functional performance [16,23,43,44,45]. Extending this evidence, the present trial provides gait-phase–resolved surface EMG data of the lower limbs following DNS, addressing a gap highlighted by Venkatesan et al. [46]. DNS is conceptualized as a motor control–oriented approach derived from developmental positions, emphasizing explicit breathing and alignment cues to promote coordinated trunk–limb stabilization [15,16,40]. While other core-stabilization programs also reduce pain and disability, the present trial contributes novel phase-specific lower-limb EMG data during gait after DNS—a measurement not typically reported in prior core-focused studies—thus expanding the evidence base without claiming superiority over alternative methods.

In terms of alignment, our clinical outcomes are consistent with Mahdieh et al. [16] and Rabieezadeh et al. [23], who reported functional improvements following DNS-based rehabilitation. At the same time, we add phase-specific lower-limb EMG information during walking, an outcome not captured in those earlier reports. With respect to movement-pattern literature, Rahimi et al. [1] documented altered hip, knee, and ankle kinematics during gait in individuals with chronic low back pain, while several other reports describe trunk motor-control deficits in this population [6,10,11,12]. The adjusted post-test differences observed at heel strike, mid-stance, and push-off suggest more task-appropriate recruitment at these phases, although these associations remain inferential because trunk muscle activity and three-dimensional biomechanics were not measured.

Finally, meta-analytic evidence supports the effectiveness of DNS in reducing pain and disability [43]; our results align with those conclusions while providing phase-specific lower-limb EMG contrasts that clarify when during the gait cycle the most prominent differences occur. Because this study did not include an active comparator, the findings should be interpreted as associative evidence for DNS rather than proof of superiority over other core-stabilization approaches. Head-to-head trials with matched dosing, larger sample sizes, and direct trunk and biomechanical assessments are warranted.

This randomized controlled trial provides preliminary evidence that an eight-week DNS program was associated with phase-specific improvements in lower-limb EMG activity and clinical outcomes in young adults with chronic low back pain. In ANCOVA models adjusted for baseline, between-group differences at post-test were evident for the vastus medialis at heel strike, tibialis anterior at mid-stance, and rectus femoris and biceps femoris at push-off, accompanied by lower pain and disability in the DNS group relative to control. These correlational findings may indicate more task-appropriate distal recruitment during gait. Confirmation of mechanisms and generalizability will require larger, multi-center trials including active comparators and direct assessments of trunk muscle function and biomechanics.

It is important to position these findings within the broader context of rehabilitative exercises. A recent meta-analysis confirmed DNS as a promising approach for reducing pain and disability [43]. Other work has shown it to be as effective as established methods like aquatic therapy [5]. Our study adds a new layer to this evidence base by demonstrating phase-specific lower-limb sEMG differences at Post-test (ANCOVA-adjusted), suggesting that the pathway by which DNS achieves positive clinical outcomes may involve improved task-appropriate recruitment in key gait phases. That said, this interpretation remains inferential, as direct core measures were not collected, and the use of a passive control does not exclude attention-related effects; future trials with active comparators and direct core assessments are warranted.

From a clinical perspective, DNS can be considered not only as a core-oriented program but as a comprehensive neurorehabilitation strategy aimed at improving motor control for functional tasks. A pragmatic template for implementation is three supervised sessions per week for eight weeks, approximately fifty minutes per session, with the main workload performed at a perceived exertion of 3 to 5 on the Borg CR10 scale. Sessions may follow a standard sequence that includes a brief warm-up, DNS developmental positions with explicit breathing and alignment cues, and a cool-down, delivered in small groups at an approximate therapist-to-participant ratio of one to six with sex-matched supervision. Eligible patients should have characteristics comparable to the study cohort, namely ambulatory young adults with non-specific chronic low back pain and no recent structured rehabilitation, with progression individualized as needed. The phase-specific findings observed here (higher vastus medialis at heel strike, lower tibialis anterior at mid-stance, and lower rectus femoris and biceps femoris at push-off) suggest task-appropriate targets for coaching and exercise selection. Clinicians may pair DNS with task-specific gait practice, use routine monitoring of symptoms and function (visual analogue scale and Oswestry Disability Index), and document adherence and session fidelity to support transfer to daily activities. Given the passive control and the absence of direct trunk-muscle measurements in this trial, these recommendations should be viewed as practice-oriented guidance rather than mechanistic prescriptions.

This study has certain limitations. Although the sample included young adults of both sexes, it was not powered to detect sex-specific effects, and small imbalances in sex distribution remained across groups. These factors limit the generalizability of the findings to other age ranges and clinical subgroups, such as highly active or older adults. Moreover, no long-term follow-up was conducted to determine whether neuromuscular adaptations persist; prior work suggests that benefits may diminish after detraining, thereby highlighting the need for sustained intervention [23]. In addition, because the control group was passive, potential attention-related (Hawthorne) effects cannot be excluded. Furthermore, we did not directly record diaphragm, transversus abdominis, or lumbar multifidus activity, which tempers causal inferences about core-mediated mechanisms. The absence of an active comparator also limits conclusions regarding the specificity of DNS relative to other interventions. Nevertheless, the ANCOVA-adjusted Post-test differences reported here provide an early signal of phase-specific modulation in lower-limb activation profiles, supporting the rationale for future trials. Therefore, future studies should recruit more diverse samples, include active comparator interventions such as conventional exercise, and implement long-term follow-up assessments. They should integrate surface EMG with 3D motion analysis to clarify how phase-specific muscle activity relates to whole-body mechanics and add direct measures of core muscle function (e.g., diaphragm/TrA/multifidus activity or IAP surrogates) to strengthen mechanistic understanding and external validity. Although this study focused on lower-limb muscle activation during gait, the specific effects of DNS on lumbo-pelvic stability during other functional tasks, such as stair climbing, lifting, or transitional movements, remain to be explored. Although assessor blinding and scripted assessments were used, nonspecific intervention effects related to attention and expectancy cannot be excluded, given the passive control design. The very high adherence and standardized small-group supervision (mean 23.6 of 24 sessions; therapist-to-participant ratio ≈ 1:6) help contextualize the between-group differences but do not eliminate these potential influences.

## 5. Conclusions

This randomized controlled trial provides preliminary evidence that an eight-week DNS program was associated with phase-specific improvements in lower-limb EMG activity and clinical outcomes in young adults with chronic low back pain. In our ANCOVA models adjusted for baseline, between-group differences at Post-test were evident for vastus medialis at heel strike, tibialis anterior at mid-stance, and for rectus femoris and biceps femoris at push-off, alongside lower pain and disability in the DNS group relative to control. These are correlational findings and may indicate more task-appropriate distal recruitment during gait. Confirmation of mechanisms and generalizability will require larger, multi-center trials with active comparators and direct assessments of trunk muscle function and biomechanics. These are correlational findings specific to a DNS protocol and do not establish superiority versus other core-stabilization approaches; comparative trials with active controls and direct trunk and biomechanical assessments are needed.

## Figures and Tables

**Table 1:** Baseline Demographic Characteristics of Participants.

Parameter	DNS (*n* = 27)	DNS (*n* = 27)	*p* (Between-Groups)
Age (years)	23.9 ± 1.3	23.6 ± 1.4	0.453
Sex (M/F)	14 (51.9%)/13 (48.1%)	14 (51.9%)/13 (48.1%)	0.579
Height (cm)	175.2 ± 5.4	174.4 ± 6.4	0.628
Weight (kg)	74.2 ± 7.2	74.2 ± 7.6	0.972
BMI (kg/m^2^)	24.1 ± 2.0	24.3 ± 2.1	0.780

Notes: Continuous variables are presented as mean ± SD; categorical variables as *n* (%). Between-group comparisons used independent-samples *t*-tests (age, height, weight, BMI) and chi-square tests (sex). No significant differences were observed at baseline (all *p* > 0.05). BMI = body mass index.

**Table 2:** Electrode Placement according to SENIAM Protocol for the Studied Muscles.

Muscle	Electrode Location	Electrode Orientation
Tibialis Anterior (TA)	At 1/3 on the line between the tip of the fibula and the tip of the medial malleolus.	Along the line between the fibula and the medial malleolus.
Gastrocnemius (GC)	On the most prominent bulge of the muscle.	Parallel to the line between the head of the fibula and the heel.
Vastus Lateralis (VL)	At 2/3 on the line from the Anterior Superior Iliac Spine (ASIS) to the lateral side of the patella.	In the direction of the muscle fibers.
Vastus Medialis (VM)	At 80% of the distance on the line between the ASIS and the joint space in front of the anterior border of the medial collateral ligament.	In an oblique direction, parallel to the muscle fibers.
Rectus Femoris (RF)	At 50% on the line from the ASIS to the superior part of the patella.	Along the line between the ASIS and the patella.
Biceps Femoris (BF)	At 50% on the line between the ischial tuberosity and the lateral epicondyle of the tibia.	Along the line between the ischial tuberosity and the fibular head.
Semitendinosus (ST)	At 50% on the line between the ischial tuberosity and the medial epicondyle of the tibia.	In the direction of the line between the ischial tuberosity and the tibia.
Gluteus Medius (GM)	At 50% on the line from the iliac crest to the greater trochanter of the femur.	Parallel to the direction of the fibers, pointing towards the greater trochanter.

**Table 3:** Clinical outcomes adjusted by ANCOVA.

Outcome	DNS (EMM ± SE)	Control (EMM ± SE)	F(1, 52)	*p*-Value	*ηp* ^2^	Levene’s *p*
VAS (0–10)	3.0 ± 0.2	6.1 ± 0.2	76.66	<0.001	0.596	0.976
ODI (%)	15.7 ± 1.5	34.3 ± 1.5	71.47	<0.001	0.579	<0.001

Notes: Adjusted means (EMMs) and standard errors are from ANCOVA with the baseline value entered as the covariate (VAS evaluated at 6.35; ODI at 35.71). Partial eta squared (*ηp*^2^) is reported for the Group effect. Covariate terms were not significant (VAS *p* = 0.560; ODI *p* = 0.359).

**Table 4:** Baseline Comparison of sEMG Muscle Activity.

Muscles	Phases	Treatment (DNS, *n* = 27) Mean ± SD	95% CI	Control (*n* = 28) Mean ± SD	95% CI	Sig. (2-Tailed)
TA	LR	88.9 ± 25.6	78.7–99.0	80.1 ± 24.6	70.5–89.6	0.200
	MC	97.5 ± 28.2	86.3–108.7	85.8 ± 24.3	76.3–95.2	0.104
	PO	90.3 ± 47.5	71.5–109.1	87.0 ± 27.5	76.3–97.7	0.752
	SW	84.2 ± 19.6	76.4–91.9	76.5 ± 21.7	68.1–85.0	0.179
GC	LR	86.4 ± 32.3	73.6–99.2	89.0 ± 24.6	79.4–98.6	0.741
	MC	85.5 ± 32.0	73.6–97.3	79.2 ± 19.6	71.8–86.7	0.387
	PO	90.4 ± 25.4	80.5–100.4	85.3 ± 30.2	73.6–97.1	0.501
	SW	84.5 ± 24.1	75.0–94.0	95.0 ± 30.4	83.4–106.7	0.163
VL	LR	64.5 ± 21.5	56.0–73.0	74.6 ± 24.4	65.9–84.8	0.110
	MC	76.1 ± 26.8	65.7–86.5	75.3 ± 24.2	66.7–84.0	0.918
	PO	71.8 ± 29.3	60.2–83.4	77.9 ± 30.2	66.2–89.6	0.449
	SW	66.1 ± 21.8	57.4–74.7	75.4 ± 30.2	63.7–87.1	0.196
VM	LR	70.2 ± 23.4	61.7–78.7	78.4 ± 21.2	70.3–86.5	0.183
	MC	75.9 ± 28.1	64.7–87.0	70.3 ± 16.0	64.0–76.5	0.365
	PO	78.4 ± 30.7	66.1–90.8	72.5 ± 24.9	62.0–82.9	0.707
	SW	68.4 ± 26.4	58.3–78.5	80.9 ± 27.2	70.2–91.5	0.091
RF	LR	67.1 ± 18.7	59.7–74.5	79.2 ± 25.4	69.4–89.1	0.051
	MC	76.2 ± 25.3	66.1–86.4	73.9 ± 16.5	67.7–80.1	0.685
	PO	74.8 ± 26.2	64.7–84.9	78.6 ± 19.5	71.5–85.7	0.542
	SW	69.3 ± 21.4	61.0–77.7	81.1 ± 28.8	70.0–92.3	0.090
BF	LR	74.1 ± 21.4	65.8–82.4	75.9 ± 27.7	65.3–86.5	0.791
	MC	86.9 ± 29.5	75.5–98.2	82.0 ± 30.6	70.4–93.6	0.552
	PO	78.9 ± 36.3	65.5–92.3	78.9 ± 26.7	68.7–89.1	0.999
	SW	76.6 ± 22.6	68.1–85.0	79.7 ± 31.6	67.7–91.6	0.679
ST	LR	82.0 ± 30.9	70.1–93.9	82.5 ± 31.8	70.2–94.9	0.947
	MC	79.0 ± 32.0	66.7–91.3	66.8 ± 24.1	57.5–76.2	0.116
	PO	86.0 ± 34.8	73.0–98.9	89.4 ± 32.3	77.3–101.6	0.703
	SW	75.8 ± 29.1	64.6–86.9	79.7 ± 23.4	70.9–88.5	0.583
GM	LR	72.9 ± 26.1	63.0–82.9	77.9 ± 30.2	66.8–89.0	0.517
	MC	74.9 ± 32.3	62.7–87.2	67.7 ± 16.8	61.2–74.2	0.302
	PO	79.9 ± 30.7	68.0–91.9	81.7 ± 20.1	74.9–88.5	0.802
	SW	67.3 ± 25.5	57.8–76.9	76.7 ± 20.9	69.7–83.8	0.141

Notes: Values are mean ± SD of baseline (%MVIC). 95% CI calculated as mean ± t·(SD/√n) with df = 26 (DNS) and df = 27 (Control). No *p* < 0.05 at baseline. TA = tibialis anterior; GC = gastrocnemius; VL = vastus lateralis; VM = vastus medialis; RF = rectus femoris; BF = biceps femoris; ST = semitendinosus; GM = gluteus medius. LR = loading response (heel strike); MC = mid-stance; PO = push-off; SW = swing.

**Table 5:** Adjusted Post-test sEMG comparisons between groups (ANCOVA).

Muscle	Gait Phase	Adjusted Mean ± SE (DNS) [95% CI]	Adjusted Mean ± SE (Control) [95% CI]	F(1, 52)	*p*-Value	*ηp* ^2^
TA	LR	88.6 ± 7.3 [73.9–103.2]	104.6 ± 7.1 [90.2–119.0]	2.406	0.127	0.044
GC		106.0 ± 13.7 [78.5–133.6]	85.5 ± 13.4 [58.4–112.6]	1.138	0.291	0.021
VL		70.8 ± 4.5 [61.6–80.0]	64.0 ± 4.5 [54.9–73.0]	1.084	0.303	0.020
VM		80.7 ± 4.3 [72.1–89.4]	68.1 ± 4.2 [59.6–76.5]	4.351	0.042 *	0.077
RF		80.1 ± 4.7 [70.5–89.8]	80.9 ± 4.7 [71.5–90.4]	0.014	0.908	0.000
BF		81.6 ± 4.8 [71.9–91.3]	85.9 ± 4.7 [76.4–95.4]	0.405	0.527	0.008
ST		84.0 ± 4.3 [75.2–92.7]	84.9 ± 4.2 [76.3–93.5]	0.025	0.875	0.000
GM		69.5 ± 4.3 [60.8–78.1]	77.8 ± 4.2 [69.3–86.3]	1.909	0.173	0.035
TA	MC	78.5 ± 4.1 [70.3–86.7]	95.2 ± 4.0 [87.2–103.3]	8.263	0.006 *	0.137
GC		79.6 ± 4.0 [71.4–87.8]	83.5 ± 4.0 [75.4–91.5]	0.442	0.509	0.008
VL		74.2 ± 4.0 [66.1–82.3]	75.7 ± 3.9 [67.8–83.6]	0.070	0.793	0.001
VM		72.1 ± 5.3 [61.3–82.9]	85.2 ± 5.2 [74.7–95.8]	3.033	0.088	0.055
RF		72.0 ± 4.5 [62.8–81.1]	83.6 ± 4.4 [74.7–92.6]	3.323	0.074	0.060
BF		77.0 ± 5.3 [66.3–87.8]	91.0 ± 5.2 [80.5–101.6]	3.493	0.067	0.063
ST		76.6 ± 4.9 [66.7–86.4]	83.3 ± 4.8 [73.6–92.9]	0.925	0.341	0.017
GM		73.8 ± 5.5 [62.7–84.9]	76.2 ± 5.4 [65.4–87.1]	0.099	0.754	0.002
TA	PO	79.2 ± 5.2 [68.6–89.8]	88.3 ± 5.1 [77.9–98.7]	1.510	0.225	0.028
GC		90.0 ± 4.5 [80.8–99.1]	86.1 ± 4.4 [77.1–95.1]	0.372	0.544	0.007
VL		68.8 ± 3.4 [61.9–75.7]	74.3 ± 3.3 [67.6–81.1]	1.314	0.257	0.025
VM		72.8 ± 4.0 [64.6–80.9]	70.0 ± 3.9 [62.0–77.9]	0.244	0.624	0.005
RF		65.6 ± 5.2 [55.1–76.2]	85.2 ± 5.1 [74.8–95.5]	7.022	0.011 *	0.119
BF		68.0 ± 3.7 [60.5–75.5]	84.9 ± 3.6 [77.6–92.3]	10.489	0.002 *	0.168
ST		76.9 ± 4.5 [67.7–86.0]	83.9 ± 4.4 [74.9–92.9]	1.211	0.276	0.023
GM		72.9 ± 4.8 [63.2–82.6]	72.4 ± 4.7 [62.8–81.9]	0.006	0.939	0.000
TA	SW	82.3 ± 4.6 [73.0–91.5]	86.3 ± 4.5 [77.2–95.4]	0.388	0.536	0.007
GC		84.0 ± 5.8 [72.3–95.7]	85.0 ± 5.7 [73.5–96.5]	0.014	0.905	0.000
VL		70.2 ± 5.4 [59.2–81.2]	78.9 ± 5.3 [68.1–89.7]	1.276	0.264	0.024
VM		66.2 ± 4.3 [57.6–74.9]	75.9 ± 4.2 [67.5–84.4]	2.516	0.119	0.046
RF		69.4 ± 4.4 [60.4–78.3]	74.5 ± 4.3 [65.8–83.3]	0.673	0.416	0.013
BF		68.0 ± 4.0 [59.9–76.1]	74.4 ± 3.9 [66.5–82.4]	1.268	0.265	0.024
ST		76.7 ± 4.3 [67.9–85.5]	80.0 ± 4.2 [71.4–88.6]	0.288	0.594	0.006
GM		75.7 ± 5.1 [65.4–85.9]	69.4 ± 5.0 [59.3–79.5]	0.756	0.389	0.014

Notes. ANCOVA models: Post-test sEMG as dependent variable; Group as fixed factor; corresponding Pre-test sEMG as covariate (SS Type III). Reported are adjusted means (±SE) from EMM at the sample mean of the covariate. Only effects of Group are shown; rows without significance (*p* > 0.05) are summarized as non-significant (examples provided). A full table with all muscles/phases is available upon request. Abbreviations: TA = tibialis anterior; GC = gastrocnemius; VL = vastus lateralis; VM = vastus medialis; RF = rectus femoris; BF = biceps femoris; ST = semitendinosus; GM = gluteus medius; LR = loading response; MC = mid-stance; PO = push-off; SW = swing. * statistical significance.

**Figure A1:**
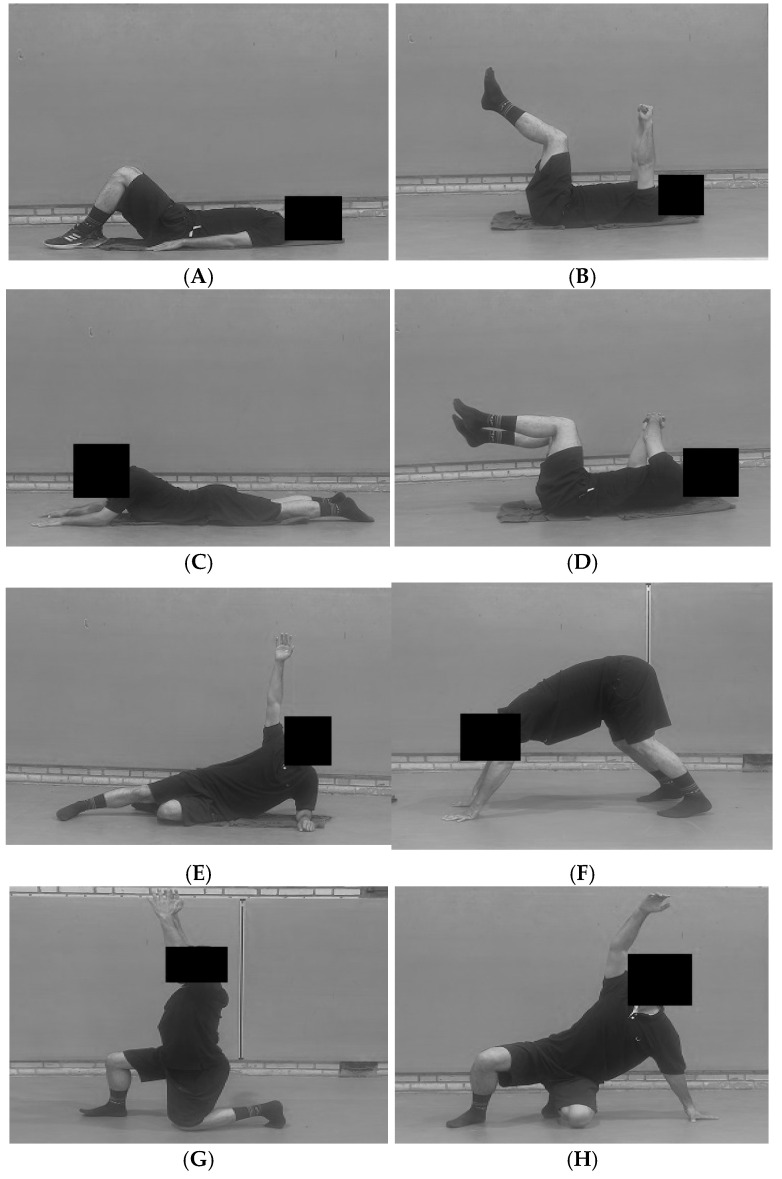
DNS exercise series and key checkpoints. Note: (**A**) Diaphragmatic breathing (supine/sitting): 360° abdominal expansion; neutral rib cage. (**B**) Baby Rock (supine 90–90): lumbopelvic neutrality; nasal breathing. (**C**) Prone on elbows: long spine; scapular control. (**D**) Rolling: trunk-led rotation without limb push-off. (**E**) Side-lying/Oblique sit: frontal-plane trunk stability; hip lift. (**F**) Tripod: contralateral support with shoulder–hip co-contraction. (**G**) High-kneeling to squat transitions: IAP maintenance through depth. (**H**) Czech Get Up (partial): smooth transitions across developmental positions.

## Data Availability

The raw data supporting the conclusions of this article will be made available by the authors on request.

## Abbreviations

The following abbreviations are used in this manuscript:

CLBP	Chronic Low Back Pain
LBP	Low Back Pain
EMG	Electromyography
DNS	Dynamic Neuromuscular Stabilization
VAS	Visual Analogue Scale
ODI	Oswestry Disability Index
MVIC	Maximal Voluntary Isometric Contraction
SENIAM	Surface Electromyography for Non-Invasive Assessment of Muscles
BMI	Body Mass Index
ANCOVA	Analysis of Covariance
SPSS	Statistical Package for the Social Sciences
G*Power	Power Analysis Software
TrA	transversus abdominis
TA	Tibialis Anterior
GC	Gastrocnemius
VL	Vastus Lateralis
VM	Vastus Medialis
RF	Rectus Femoris
ST	Semitendinosus
LR	Loading Response
MC	Mid-Stance
PO	Push-Off
SW	Swing
CGU	Czech Get Up

## Appendix A

### Appendix A.1

**Table A1:** The 8-Week DNS Program.

Exercise/Developmental Position	Week(s)	Description/Key Focus	Sets × Reps/Duration
Diaphragmatic Breathing	1–2	In supine and sitting positions, focus on 360-degree expansion of the abdominal wall and lower ribcage without chest elevation. Establish ideal Intra-Abdominal Pressure (IAP) regulation.	3 Sets × 10 Breaths
Baby Rock (Supine 90–90)		Lie supine with hips and knees at 90 degrees. Maintain a stable lumbar spine (low back flat on the floor) and controlled breathing.	3 Sets × 30-s Hold
Prone on Elbows	3–4	From a prone position, lift the torso onto the elbows. Focus on maintaining a long, neutral spine and avoid scapular winging or excessive lumbar lordosis.	3 Sets × 30-s Hold
Rolling		Initiate rotation from the core (using IAP and obliques), not by pushing with arms or legs. Movement should be smooth from supine to prone and back.	2 Sets × 8–10 Rolls per side
Side Lying & Oblique Sit	5–6	Stabilize the trunk in the frontal plane. Progress from lying on the side to supporting on one hand in the “oblique sit” position, lifting the hip off the floor.	3 Sets × 20–30-s Hold per side
Tripod Position		Support on two hands and one foot/knee. Develop contralateral support patterns, focusing on co-contraction of the supporting shoulder and hip stabilizers.	3 Sets × 20-s Hold per side
Kneeling to Squat	7–8	Practice transitioning from a high-kneeling position to a deep squat while maintaining an upright torso, neutral spine, and stable IAP.	2 Sets × 10–12 Reps
Czech Get Up (CGU)—Partial		Practice smooth transitions through the foundational DNS positions (e.g., from supine to oblique sit to tripod). Focus on the quality and control of each transition.	2 Sets × 3–5 Transitions per side

Note: The exercises listed represent the key developmental positions introduced and practiced within each phase. A typical ~40 min main session consists of a selection of these drills, progressing in complexity as outlined.
